# A comparative study of a theoretical neural net model with MEG data from epileptic patients and normal individuals

**DOI:** 10.1186/1742-4682-2-37

**Published:** 2005-09-07

**Authors:** A Kotini, P Anninos, AN Anastasiadis, D Tamiolakis

**Affiliations:** 1 Laboratory of Medical Physics, Medical School, Democritus University of Thrace, University Campus, Alex/polis, 68100, Greece; 2 General Hospital of Chania, Crete, Greece

**Keywords:** Poisson distribution, Gauss distribution, MEG

## Abstract

**Objective:**

The aim of this study was to compare a theoretical neural net model with MEG data from epileptic patients and normal individuals.

**Methods:**

Our experimental study population included 10 epilepsy sufferers and 10 healthy subjects. The recordings were obtained with a one-channel biomagnetometer SQUID in a magnetically shielded room.

**Results:**

Using the method of x^2^-fitting it was found that the MEG amplitudes in epileptic patients and normal subjects had Poisson and Gauss distributions respectively. The Poisson connectivity derived from the theoretical neural model represents the state of epilepsy, whereas the Gauss connectivity represents normal behavior. The MEG data obtained from epileptic areas had higher amplitudes than the MEG from normal regions and were comparable with the theoretical magnetic fields from Poisson and Gauss distributions. Furthermore, the magnetic field derived from the theoretical model had amplitudes in the same order as the recorded MEG from the 20 participants.

**Conclusion:**

The approximation of the theoretical neural net model with real MEG data provides information about the structure of the brain function in epileptic and normal states encouraging further studies to be conducted.

## Introduction

Epilepsy is a disorder involving recurrent unprovoked seizures: episodes of abnormally synchronized and high-frequency firing of neurons in the brain that result in abnormal behaviors or experiences. This is a fairly common disorder, affecting close to 1% of the population. The lifetime risk of having a seizure is even higher, with estimates ranging from 10 to 15% of the population. Epilepsy can be caused by genetic, structural, metabolic or other abnormalities. Epileptic disorders can be generalized, partial (focal) or undetermined. A primary generalized seizure starts as a disturbance in both hemispheres synchronously, without evidence of a localized onset. Partial forms of epilepsy start in a focal area of the brain and may remain localized without alteration of consciousness.

MEG is a noninvasive imaging technique, applicable to the human brain with temporal resolution approximately ~1 ms [1]. Several authors during the last decade have demonstrated the importance of MEG in the investigation of normal and pathological brain conditions [2,3]. The major advantage of MEG over electroencephalography (EEG) is that MEG has higher localization accuracy. This is because the different structures of the head (brain, liquor cerebrospinalis, skull and scalp) influence the magnetic fields less than the volume current flow that causes the EEG. Also, MEG is reference free, so that the localization of sources with a given precision is easier for MEG than it is for EEG [4].

The goal of this study is to compare the theoretical model that follows Poisson or Gauss distributed connectivity [5-12] with experimental MEG data from epileptic patients and healthy volunteers.

## Methods

### Description of the model

Neural nets are assumed to be constructed of discrete sets of randomly interconnected neurons of similar structure and function. The neural connections are set up by means of chemical markers carried by the individual cells. Thus, the neural population of the net is treated as a set of subpopulations of neurons, each of them characterized by a specific chemical marker. We attribute the appropriate Poisson or Gauss distribution law to each subsystem to describe connectivity.

The elementary unit, the neuron, is bistable. It can be either in the resting or in an active (firing) state. The transition from the resting to the firing state occurs when the sum of postsynaptic potentials (PSPs) arriving at the cell exceeds the firing threshold θ of the neuron. PSPs may be excitatory (EPSPs) or inhibitory (IPSPs), shifting the membrane potential closer to or further away from θ, respectively. Each neuron may carry an electrical potential of a few millivolts, which it passes on to the neurons to which it is connected.

In this model, a net with N markers is assumed to be constructed of A formal neurons. A fraction h (0<h<l) of these are inhibitory with all the axon branches generating IPSPs, while the rest are excitatory with all their axon branches generating EPSPs. Each neuron receives, on average, μ^+ ^EPSPs and μ^- ^IPSPs. The size of the PSP produced by an excitatory (inhibitory) unit is K^+ ^(K^-^). The neurons are also characterized by the absolute refractory period and the synaptic delay τ. If a neuron fires at time t, it produces the appropriate PSP after a fixed time interval τ, the synaptic delay. PSPs arriving at a neuron are summed instantly, and if this sum is greater or equal to θ, then the neuron will fire immediately, otherwise it will be idle. PSPs (if below θ) will persist with or without decrement for a period called the summation time, which is assumed to be less than the synaptic delay. Firing is momentary and causes the neuron to be insensitive to further stimulation for a time interval called the (absolute) refractory period [5-12].

The mathematical formalism of this study is based on the equations for the expectation values of the activity derived in previous papers [5-12]. A brief mathematical analysis for each case is given below.

#### a) Expectation value of neural activity in noiseless and noisy neural nets with Poisson distributed connectivities

Following the assumptions of previous papers it was shown that the expectation value of the neural activity <α_n+1_> at t = (n+1) τ, i.e. the average value of α_n+1 _generated by a collection of netlets with identical statistical parameters (μ^+^, μ^-^, h, K^+^, K^-^, A, θ) and the same α_n _at t = nτ, is given by:

<α_n+1_> = (1-α_n_) P (α_n_, θ)     (1)

where P(α_n_, θ) is the probability that a neuron receives post synaptic potentials (PSPs) exceeding its threshold at time t = (n+1)τ. Thus:



Here P_l _and Q_m _are the probabilities that a neuron will receive l and m EPSPs and IPSPs respectively, and are given by (3):

P_I _= exp (-α_n _(1-h) μ^+^) (α_n _(1-h) μ^+^)^l^/l!

Q_m _= exp (-α_n _h μ^-^) (α_n _hμ^-^)^m^/m!     (3)

In addition, the upper limits in the double sum m_max _and l_max _are given by (4):

l_max _= A α_n _(1-h) μ^+^

m_max _= Aα_n_hμ^- ^    (4)

Taking into account equations (2) and (3), equation (1) takes the form:



Similarly for Poisson nets with noise: if P_l _and Q_m _are the probabilities that a given neuron receives I EPSPs and m IPSPs at time t = (n+1)τ, they are given by equation (3). But if T_δ _(θ) is the probability that the instantaneous threshold value is θ or less than θ, this is given by (6):



Therefore the firing probability per neuron is then given by (7):



where l_max _and m_max _are given by equation (4).

Finally, the expectation value of the activity is given by (8):

<α_n+1_> = (1-α_n_) P (α_n_, θ)     (8)

#### b) Expectation value of neural activity in neural nets with Poisson distributed connectivities with chemical markers and noise

Similarly, the expectation value of the activity <α_n+1_> for an isolated neural net with two chemical markers a and b is given by (9):



where P_I_, Q_i_, P'_l_, Q'_i'_, are the probabilities that a given neuron will receive l EPSPs, i IPSPs or l'-EPSPs, i'-IPSPs, at time t = (n+1)τ in the subsystems a or b respectively. These probabilities are given by (10):

P_l _= exp (-α_n _μ_a_^+ ^(1-h_a_) m_a_) (-α_n _μ_a_^+ ^(1-h_a_) m_a_)^l^/l!

Q_i _= exp (-α_n _μ_a_^- ^h_a _m_a_) (-α_n _μ_a_^- ^h_a _m_a_)^i^/i!

P'_l' _= exp (-α_n _μ_b_^+ ^(1-h_b_) (1-m_a_)) (-α_n _μ_b_^+ ^(1-h_b_) (1-m_a_))^l'^/l'!

Q'_i' _= exp (-α_n _μ_b_^- ^h_b _(1-m_a_)) (-α_n _μ_b_^- ^h_b _(1-m_a_))^i'^/i'!     (10)

The upper limits in the sums in equation (9) are given by (11):

l_max _= A α_n _μ_a_^+ ^(1-h_a_) m_a_

l_max' _= A α_n _μ_b_^+ ^(1-h_b_) (1-m_a_)

i_max _= A α_n _μ_a_^- ^h_a _m_a_

i_max' _= A α_n _μ_b_^- ^h_b _(1-m_a_)     (11)

Finally,  (θ_a_) and  (θ_b_) are defined as the probabilities that the instantaneous neural thresholds are equal to or less than θ_a _and θ_b _in subsystems a and b respectively and are given by (12):



#### b) Expectation value of neural activity in neural nets with Gaussian connectivities in the absence of chemical markers

Let the total PSP of a neuron at t = (n+1)τ be given by:

e_n+1 _= lK^+ ^+ mK^- ^    (13)

where l and m are the numbers of EPSPs and IPSPs respectively. If both l and m are large, their distributions may be approximated by Gaussian distributions about their respective average values  and . The distribution of e_n+1 _is therefore also normal, since the probabilities for l and m are mutually independent, and its variance is the sum of the variances of l and m. Therefore the average PSP will be given by (14):



where K = [μ^+ ^(1-h) K^+ ^+ μ^-^h K^-^]     (14)

The variance of e_n+1_, call it , is then given by (15):

 = α_n _[μ^+ ^(1-h) (K^+^)^2 ^+ μ^-^h (K^-^)^2^]     (15)

The probability that the PSP exceeds a threshold  now becomes:



Equation (16) in conjunction with equation (1) gives values for <α_n+1_> at t = (n+1)τ.

Let T(θ') be the probability that the instantaneous threshold of a neuron is θ' or less than θ'. This is given by (17):



Here δ is the standard deviation of the Gaussian distribution of the noise. Finally, the probability that a neuron will receive PSPs that will exceed the threshold at time t = (n+1)τ is given by (18):





Since l and m are very large numbers, the double sum can be approximated by  and therefore:



Then the expectation value of <α_n+1_> of the activity at time t = (n+1)τ will be:

<α_n+1_> = (1-α_n_) P(α_n_, δ_n+1_, δ)     (20)

#### c) Expectation value of neural activity in noisy neural nets with chemical markers and Gaussian distributed connectivities

In a neural netlet of A neurons with two chemical markers a and b, let the fractional numbers corresponding to each chemical marker be m_a _and m_b_, and the fractions of inhibitory neurons for each chemical marker be h_a _and h_b_, respectively. Also, let α_n_A be the active neurons in the netlet at t = nτ. Then at t = (n+1)τ the numbers of EPSPs and IPSPs that will appear in the subsystems with a and b markers will be:

l_a _= A α_n _μ_a_^+ ^(1-h_a_) m_a_

i_a _= A α_n _μ_a_^- ^h_a _m_a_

l_b _= A α_n _μ_b_^+ ^(1-h_b_) m_b_

i_b _= A α_n _μ_b_^- ^h_b _m_b _    (21)

On the average, the numbers of EPSPs and IPSPs that appear per neuron in subnets with a and b markers will be:

 = α_n _μ_a_^+ ^(1-h_a_) m_a_

 = α_n _μ_a_^- ^h_a _m_a_

 = α_n _μ_b_^+ ^(1-h_b_) m_b_

 = α_n _μ_b_^- ^h_b _m_b _    (22)

As stated in our previous papers [5-12] the total PSP input to a neuron with a and b markers at t = (n+1)τ will be given by (23):

e_a,n+1 _= l_a_K^+ ^+ i_a_K^-^

e_b,n+1 _= l_b_K^+ ^+ i_b_K^- ^    (23)

(Here it is assumed that )

If the quantities l_a_, l_b_, i_a _and i_b _are sufficiently large, their distributions may be approximated by Gaussian distributions about their average values, given by (22). Then the average PSPs for the two markers a and b will be given by (24):



and their variances will be given by (25):



Therefore the probability that a neuron with marker a or b will receive a certain number of EPSPs or IPSPs that will shift the membrane potential closer to or further away from the instantaneous threshold will be given by (26):



where:



Thus, the probabilities  and  that the instantaneous threshold of a neuron in subsystems a and b is equal to or less than  or  will be given by (28):



Consequently, as stated in our previous paper [8], the firing probabilities P(α_n_, δ_n+1_, δ_a_) and P'(α_n_, δ_n+1_, δ_b_) that a neuron in subpopulations a and b, respectively, will receive PSPs exceeding threshold at time t = (n+1)τ will be given by (29):



Since the quantities l_a_, i_a_, l_b _and i_b _are sufficiently large, the double sum in equations (29) will be substituted by the probabilities of the average values of l_a_, i_a _and l_b_, i_b _for each marker a and b and will be given by (30):



Then according to our previous papers [5-12], the expectation value of activity in this netlet with two markers a and b at time t = (n+1)τ will be given by (31):



The general case for an isolated noisy net with N markers m_1_, m_2_,..., m_N_, where m_i _is the fraction of neurons with the i^th ^marker, is described by an equation analogous to the equation for two markers (31). This general equation for such a netlet at time t = (n+1)τ is:



### Theoretical analysis

#### The electromagnetic fields generated in neural networks with Poisson or Gauss connectivities

Let us consider an isolated neural network with structural parameters A, μ^+^, μ^- ^and h, and initial activity α_n _at time t = nτ. The potential generated in this network due to this initial activity will be equal to the summation of all the PSPs [7] and will be given by (33):

V_n _= α_n _(A μ^+ ^(1-h) - A μ^-^h)     (33)

Similarly, the potential generated by the neural activity α_n+1 _at the next time interval t = (n+1)τ will be given by (34):

V_n+1 _= α_n+1 _(A μ^+ ^(1-h) - A μ^-^h)     (34)

By combining equations (33) and (34) and assuming spherical brain symmetry, the potential difference ΔV can be obtained. As is known from classical physics, this generates a magnetic field B_n _given by (35):



Choosing Δt = 1 ms, the above equation takes the following form:



where μ_o _and ε_o _are the magnetic permeability and dielectric constant of the medium respectively.

When the neural network is characterized by two chemical markers a and b, the potentials created at the synapses of the neurons with the a and b markers will be given by (37):

V_na _= α_n _(A μ_a_^+ ^(1-h_a_) m_a _- A μ_a_^- ^h_a _m_a_)

V_nb _= α_n _(A μ_b_^+ ^(1-h_b_) m_b _- A μ_b_^- ^h_b _m_b_)     (37)

On the other hand, the total voltages created at the synapses of the neurons at times t = nτ and t = (n+1)τ will be given by (38):

V_n _= V_na _+ V_nb _= α_n _A [(μ_a_^+ ^(1-h_a_) m_a _+ μ_b_^+ ^(1-h_b_) m_b_) - (μ_a_^- ^h_a _m_a _+ μ_b_^- ^h_b _m_b_)]

V_n+1 _= α_n+1 _A [(μ_a_^+ ^(1-h_a_) m_a _+ μ_b_^+ ^(1-h_b_) m_b_) - (μ_a_^- ^h_a _m_a _+ μ_b_^- ^h_b _m_b_)]     (38)

Therefore the potential difference between these two time intervals, taking into account equations (38), is given by (39):

ΔV = V_n+1 _- V_n _= (α_n+1 _- α_n_) A [(μ_a_^+ ^(1-h_a_) m_a _+ μ_b_^+ ^(1-h_b_) m_b_) - (μ_a_^- ^h_a _m_a _+ μ_b_^- ^h_b _m_b_)]     (39)

Thus, as stated previously, this potential difference will create a magnetic field B_n_, which is given by (40):



where the neural activity α_n+1 _refers to a Poisson or Gauss distribution of connectivities as given in the previous section.

In the general case, where the neural net has N chemical markers, equation (40) takes the form:



##### Experimental procedure

We compared the theoretical results with the experimental findings obtained using MEG measurements from 10 epileptic patients and 10 healthy volunteers. Informed consent for the methodology and the aim of the study was obtained from all participants prior to the procedure.

Biomagnetic measurements were performed using a second order gradiometer SQUID (Model 601, Biomagnetic Technologies Inc.), which was located in a magnetically shielded room with low magnetic noise. The MEG recordings were performed after positioning the SQUID sensor 3 mm above the scalp of each patient using a reference system. This system is based on the International 10–20 Electrode Placement System [13] and uses any one of the standard EEG recording positions as its origin; in this study we used the P3, P4, T3, T4, F3, and F4 recording positions [14-16]. Around the origin (T3 or T4 for temporal lobes) a rectangular 32-point matrix was used (4 rows × 8 columns, equidistantly spaced in a 4.5 cm × 10.5 cm rectangle) for positioning of the SQUID [14-16]. The MEG was recorded from each temporal lobe at each of the 32 matrix points of the scalp for 32 s and was band-pass filtered with cut-off frequencies of 0.1 and 60 Hz. The MEG recordings were digitized using a 12 bit precision analog-to-digital converter with a sampling frequency of 256 Hz, and were stored in a PC peripheral memory for off-line Fourier statistical analysis. The method, by its nature (i.e. temporal and spatial averaging), eliminates short-term abnormal artifacts in any cortical area, while it retains long-lasting localized activation phenomena. We used the x^2 ^– fitting method to analyze the MEG data [17].

This method was based on the following equation (42):



where:

Q_i_: is the number of elements in the i^th ^interval of the normalized MEG histogram

T_i_: is the number of elements in the i^th ^interval of the normal distribution with the same mean value and standard deviation as the normalized MEG histogram

k: is the number of intervals

n = k-1: the degrees of freedom of the system

In our case n = 7 and the critical value for distinguishing the Poisson from the Gauss distribution was 14.1 (x_cr_^2 ^= 14.1). If the estimated value of the x^2 ^was greater than 14.1, the distribution was Poisson; otherwise it was Gauss.

## Results

Using the x^2^-fitting method it was found that the MEG recordings from epileptic patients had Poisson distributions whereas those from normal subjects had Gauss distributions. The Poisson connectivity derived from the theoretical model represents the state of epilepsy, whereas the Gauss connectivity represents normal behavior. The magnetic field derived from the theoretical model was approximately in the same order as the recorded MEG in both conditions. Furthermore, the MEG data obtained from epileptic areas had higher amplitudes than those from normal regions and were comparable with the theoretical magnetic fields from Poisson and Gauss distributions.

**Figure 1:**
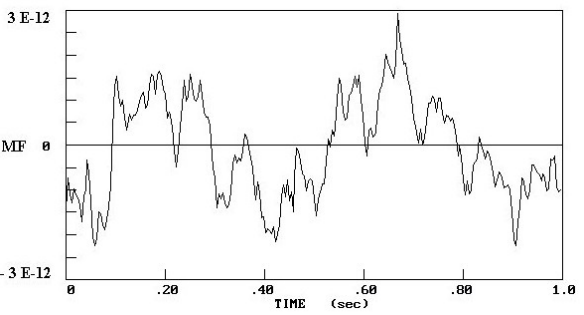
The MEG recorded from an epileptic patient over an interval of 1 s duration. The x-axis represents the time sequence and the y-axis the magnetic field.

**Figure 2:**
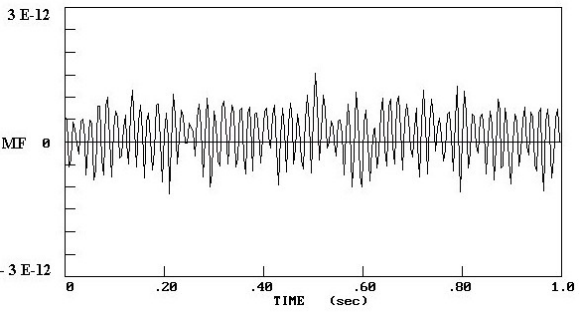
The MEG recorded from a healthy subject over an interval of 1 s duration. The x-axis represents the time sequence and the y-axis the magnetic field.

Figure 1 shows the MEG recorded from an epileptic patient; figure 2 illustrates the MEG recorded from a healthy volunteer.

**Figure 3:**
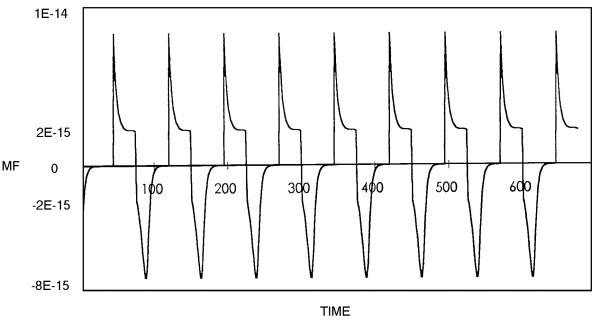
The magnetic field derived from the theoretical model with Poisson distribution. The x-axis represents the time sequence and the y-axis the magnetic field. Parameters: m_a _= 0.6, θ_a _= 5,  = 15, h_a _= 0; m_b _= 0.2, θ_b _= 4,  = 192, h_b _= 0.01; m_c _= 0.1, θ_c _= 3,  = 34, h_c _= 0.01; m_d _= 0.1, θ_d _= 3,  = 32, h_d _= 0; K^± ^= 1.

**Figure 4:**
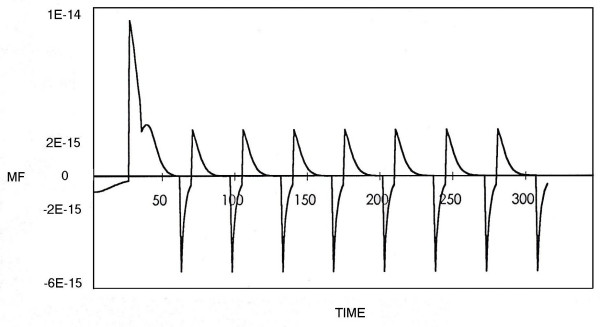
The magnetic field derived from the theoretical model with Gauss distribution. The x-axis represents the time sequence and the y-axis the magnetic field. Parameters: m_a _= 0.7, θ_a _= 6,  = 14, h_a _= 0; m_b _= 0.08, θ_b _= 4,  = 240, h_b _= 0.02; m_c _= 0.02, θ_c _= 4,  = 400, h_c _= 0.0; m_d _= 0.1, θ_d _= 4,  = 337, h_d _= 0; m_e _= 0.1, θ_e _= 4,  = 294, h_e _= 0.03; K^± ^= 1.

Figures 3 and 4 show the magnetic fields derived from the theoretical model with Poisson and Gauss distributions respectively.

## Discussion

Over the past three decades, neural nets have been intensively studied from several points of view. An area of considerable importance is that of biological nets, i.e. models of nets designed to imitate the structures and functions of human and other living brains and thus enhance our understanding of learning, memory, understanding etc. Widely used models include the pioneering work of McCulloch and Pitts [18], which treats assemblies of neurons as logical decision elements, the mathematical formalism of Caianiello [19] using the "neuronic equation", and probabilistic neural structures [5,6] that monitor the net activity, i.e. the fraction of neurons that become active per unit time. All these models have had a measure of success in improving our understanding of functions such as those mentioned above.

The effect of structure on function and dynamic behavior in neural nets has been also a subject of considerable interest in recent years. In the so-called probabilistic nets we have an assembly comprising a large number of neurons, randomly positioned in space, that have only partial connectivity; i.e. each neuron is connected to only a very small fraction of the total number of neurons in the system, randomly chosen. The principal idea is that this connectivity is given by the binomial distribution. In earlier work, probabilistic neural nets were investigated using Poisson or Gauss distributions of interneuronal connectivity; the main conclusion was that when a neuron was connected to a relatively small number of units, a Poisson distribution law was appropriate but if it was connected to a large number of units then a Gaussian law was a fairly good approximation [10-12]. Thus, Poisson neuronal nets may be viewed as approximately Gaussian whenever the number of synaptic connections is relative large.

In this study we measured the MEG of epileptic patients and normal subjects in order to compare the theoretical neural net model [10-12] with real data. Analyzing the MEG data by x^2^-fitting revealed that the MEG recordings from epileptic areas had Poisson distributions [17]. This finding is consistent with the correspondence between Poisson distributions and low numbers of internal neural connections, and with the synchronization of neural activity during an epileptic seizure [20,21]. Moreover, the MEG recordings from epileptic areas showed higher amplitudes than those from normal regions, comparable with the results from the theoretical neural model with Poisson and Gauss distributions respectively (Figs. 1, 2, 3, 4).

If a nerve cell is characterized by a given firing threshold which, when exceeded, results in spike discharge, two anatomical situations can be contrasted: one in which only a few synaptic contacts reach the cell in question, and a second in which the cell receives a large number of synaptic inputs. Suppose, in either case, that firing is dependent on the simultaneous excitation of a certain percentage of the total synaptic input (assuming that the ratio of excitatory and inhibitory synapses is the same in both situations so that the inhibitory inputs may be disregarded for the moment). Then it is clear that firing in neurons with a large number of synaptic inputs would require the synchronized activation of a substantial number of synapses; whereas in neurons with few synapses, firing may ensue even from a single excitatory synapse. Thus, a system in which neurons receive small numbers of synaptic connections is likely to exhibit a less "controlled" pattern of activity – and also "spontaneous" discharges [22]. The inverse problem in MEG measurements is the search for unknown sources by analysis of the measured field data. To handle this task one must first study the forward problem, i.e. how the magnetic field and the electrical potential arise from a known source. For practical purposes one also has to choose appropriate models for the source and the biological object as a conductor. Sarvas [23] described basic mathematical and physical concepts relevant to the forward and inverse problems and discussed some new approaches. Especially, he described the forward problem for both homogenous and inhomogenous media. He referred to the Geselowitz's formulae and presented a surface integral equation to handle a piecewise homogenous conductor and a horizontally layered medium. Furthermore, he discussed the non-uniqueness of the solution of the magnetic inverse problem and studied the difficulty caused by the contribution of the electric potential to the magnetic field outside the conductor.

The Poisson distribution corresponds to epileptic areas and the Gauss distribution to normal regions. The approximation of the theoretical neural net model to real MEG data provides a mathematical approach to the structure of brain function and indicates the need for further studies.

## Appendix

The subscript i is a marker label and indicates the properties of a subpopulation in the netlet characterized by the i^th ^marker.

### Structural parameters of the neural net

τ Synaptic delay

A Total number of neurons in the netlet

h_i _Fraction of inhibitory neurons

 The average number of neurons receiving excitatory postsynaptic potentials (EPSPs) from one excitatory neuron

 The average number of neurons receiving inhibitory postsynaptic potentials (IPSPs) from one inhibitory neuron

 The size of PSP produced by an excitatory neuron of the netlet

 The size of PSP produced by an inhibitory neuron of the netlet

m_i _Fractions of neurons carrying the i^th ^marker in the netlet

θ_i _Firing thresholds of neurons

### Statistical parameters

δ_i _Standard deviation of the Gaussian distribution of the neural firing thresholds in the i^th ^subpopulation

### Dynamical parameters

n An integer giving the number of elapsed synaptic delays

α_n _The activity, i.e. the fractional number of active neurons in the netlet at time t = nτ
